# Maternal Concerns in Home Care for the Premature Newborn: An Integrative Review

**DOI:** 10.1590/0034-7167-2022-0769

**Published:** 2023-12-04

**Authors:** Thaís Emanuele da Conceição, Maria Helena do Nascimento Souza, Rafael Braga Esteves, Patrícia Lima Pereira Peres, Donatella Valente, Antonella Nespoli

**Affiliations:** I Universidade Federal do Rio de Janeiro. Rio de Janeiro, Rio de Janeiro, Brazil; II Faculdade Fleming Cerquilho. Cerquilho, São Paulo. Brazil; III Universidade Estadual do Rio de Janeiro. Rio de Janeiro, Rio de Janeiro, Brazil; IV Sapienza Università di Roma. Lácio, Roma, Italy; V Universidade de Milano-Bicocca. Milão, Lombardia, Italy

**Keywords:** Infant Care, Infant Premature, Patient Discharge, Home Nursing, Nursing Care, Cuidado del Lactante, Recien Nascido Prematuro, Alta del Paciente, Atención Domiciliaria de Salud, Atención de Enfermería, Cuidado do Lactente, Recém-Nascido Prematuro, Alta do Paciente, Assistência Domiciliar, Cuidados de Enfermagem

## Abstract

**Objective:**

To identify and analyze the scientific literature, both national and international, concerning the primary maternal concerns about caring for premature newborns at home.

**Methods:**

This integrative review is based on the guiding question: “What scientific publications from 2012 to 2021 address maternal concerns about the care of premature newborns at home?”. Searches were conducted in the electronic databases: Embase, Medline, Web of Science, Lilacs, Scielo, and Cochrane Library.

**Results:**

A total of 21 articles were identified. The qualitative analysis showed that maternal concerns pertained to breastfeeding, hygiene, sunbathing practices, managing infant colic, identifying signs, symptoms, and clinical changes, temperature control, and the third phase of the kangaroo method.

**Conclusions:**

Maternal uncertainties underscore the importance of enhancing strategies focused on supporting families and ensuring continued care for neonates at home.

## RESUMO

**Objetivo:**

Identificar e analisar as produções científicas disponíveis na literatura nacional e internacional sobre as principais dúvidas maternas no cuidado ao recém-nascido prematuro em domicílio.

**Métodos:**

Estudo de revisão integrativa, fundamentado na questão norteadora: “Quais são as produções científicas publicadas no período de 2012 a 2021 sobre as dúvidas maternas no cuidado ao recém-nascido prematuro em domicílio?”. As buscas foram realizadas nas bases eletrônicas: *Embase, Medline, Web of Science, Lilacs, Scielo e Cochrane Library.*

**Resultados:**

Foram identificados 21 artigos. A análise qualitativa revelou que as dúvidas maternas estavam relacionadas ao aleitamento materno, higiene, prática de banho de sol, conduta em relação à cólica do bebê, identificação de sinais, sintomas e alterações clínicas, controle térmico e à terceira etapa do método canguru.

**Conclusões:**

As incertezas maternas destacaram a importância do aprimoramento das estratégias voltadas para o apoio à família e à continuidade do cuidado ao neonato em domicílio.

## RESUMEN

**Objetivo:**

Identificar y analizar las producciones científicas disponibles en la literatura nacional e internacional sobre las principales inquietudes maternas en el cuidado del recién nacido prematuro en el hogar.

**Métodos:**

Estudio de revisión integradora, fundamentado en la pregunta guía: “¿Cuáles son las producciones científicas publicadas en el período de 2012 a 2021 sobre las inquietudes maternas en el cuidado del recién nacido prematuro en el hogar?”. Las búsquedas se realizaron en las bases electrónicas: Embase, Medline, Web of Science, Lilacs, Scielo y Cochrane Library.

**Resultados:**

Se identificaron 21 artículos. El análisis cualitativo reveló que las inquietudes maternas estaban relacionadas con la lactancia materna, higiene, práctica de baños de sol, comportamiento ante el cólico del bebé, identificación de signos, síntomas y cambios clínicos, control térmico y a la tercera etapa del método canguro.

**Conclusiones:**

Las incertidumbres maternas resaltaron la importancia de mejorar las estrategias dirigidas al apoyo familiar y a la continuidad del cuidado del neonato en el hogar.

## INTRODUCTION

Prematurity is regarded as a global public health issue. Births that occur between the 20th and 37th gestational weeks are among the main risk factors for infant morbidity and mortality, especially neonatal mortality. Moreover, this results in a demand for more complex clinical care and increases in family expenses and health service costs^(1-2)^.

In developed countries, the high prevalence of prematurity is linked to the use of assisted reproduction techniques, multiple pregnancies, and maternal age exceeding 34 years. Conversely, in developing countries, the primary causes of prematurity are associated with poor socioeconomic conditions, lack of quality healthcare, complications during pregnancy, and childbirth issues^(3-4)^. In this context, in Brazil in 2019, complications during childbirth accounted for 11.08% of prematurity cases and continue to be one of the leading causes of neonatal deaths^(5)^.

Moreover, premature newborns need continuous care due to functional limitations, dependence on medications, special feeding requirements, the necessity for technological devices, and direct assistance from healthcare professionals. This situation calls for adequate information, social support, and lifestyle adjustments for families^(3-4)^.

In fact, many mothers of premature newborns first encounter the Neonatal Intensive Care Unit (NICU) environment before experiencing their family setting. This sequence can jeopardize the mother-infant bond post-discharge and delay the monitoring of growth and development by primary healthcare professionals during the First Integral Health Week, as proposed by the Ministry of Health^(3-6)^.

Furthermore, hospital staff often become so engrossed in the care of the baby that they neglect to include the family in the care process and do not establish a welcoming environment. This approach can result in care that doesn’t emphasize promoting and strengthening the family bond with the newborn or integrating with primary care networks to ensure consistent support and monitoring^(7)^.

Hence, professionals who provide direct care should alleviate the negative impacts of the hospital setting for mothers/guardians. They should encourage these primary caregivers to participate actively in care moments while always respecting the newborn’s clinical stability. This involvement enhances bonding opportunities and bolsters the mother-child relationship^(8)^.

Existing literature emphasizes the significance of guidance for preparing families of newborns, as given by health professionals, during discharge from an intensive care or neonatal unit^(9-12)^. In line with this, a study conducted in Paraná, Brazil, underscored the necessity to understand maternal emotions during the hospitalization of the premature newborn and after discharge. This focus is crucial as emotions such as fear, insecurity, and concern can influence home care^(9)^. Concurrently, a study in China stressed the value of healthcare professionals offering care and guidance to families of premature infants, drawing insights from listening to parents’ experiences and concerns^(11)^. However, a knowledge gap remains regarding the specific need’s mothers of premature infants grapple with when transitioning to a new life phase and regarding the continuity of child care at home.

## OBJECTIVE

To identify and analyze scientific papers available in both national and international literature regarding the main maternal concerns when caring for premature newborns at home.

## METHODS

### Ethical Aspects

This study is a literature review, using secondary data extracted from articles published in scientific journals. As such, it was not submitted to a research ethics committee.

### Study Type

This is an integrative literature review of an exploratory nature. Its methodological approach includes both experimental and non-experimental studies, combining data from theoretical and empirical literature with the aim of understanding the subject under analysis^(13)^. Thus, the study sought to gather publications on the care of premature newborns at home.

For the selection of texts, the inclusion criteria were: studies published in article format related to the research theme, available in the English, Spanish, and Portuguese languages, and published in the chosen databases. Editorial articles, expanded abstracts, and letters to the editor were excluded. Duplicate documents were counted only once, and those not related to the theme were excluded.

The time frame considered for the research was from 2012 to 2021, during which no literature review on the topic was identified, taking into account all scientific production and technological advances of the last decade.

To carry out this research, the following methodological steps were taken: identification of the theme, formulation of the guiding question, establishment of inclusion criteria, literature search, categorization of studies, interpretation of results, and synthesis of knowledge^(14)^. The “Population, Interest, and Context” (PICo)^(15)^ strategy was employed, where “P” refers to the mother of the premature newborn, “I” to maternal concerns, and “Co” to the home environment.

Therefore, the question guiding this study was: “What are the scientific papers published from 2012 to 2021 regarding maternal concerns in the care of premature newborns at home?”.

### Research and Selection Strategy

Articles were identified through searches in 6 electronic databases: Excerpta Medica Database (EMBASE), Medical Literature Analysis and Retrieval System Online (Medline) via PubMed, Web of Science via the CAPES Journals Portal, Latin American Literature in Health Sciences (LILACS), Scientific Electronic Library Online (SCIELO), and Cochrane Library. Searches were conducted in electronic databases between January and May of 2022. Search strategies included controlled descriptors and their synonyms researched in English from Health Science Descriptors (DeCS) and Medical Subject Heading (MeSH) via PubMed, non-controlled descriptors, all connected by the Boolean operators AND and OR, as well as symbols like truncation, depending on the characteristics of each of the searched databases. The finalized search strategies for each database are presented in Chart 1.

**Chart 1:** Search strategies performed in databases, 2022

Database	Search Strategy/Syntax	Sample (n)
EMBASE	(‘home care’ OR (care AND domiciliary)) AND (premature OR (newborn AND premature))	01
Web of Science	Premature newborn (All fields) and postnatal care (All fields) or patient discharge (All fields) and home care (All fields) and maternal behavior (All fields) and professional-family relations (All fields)	11
LILACS	(“newborn premature” OR “newborn”) AND (“postnatal” OR “maternal behavior”) AND ( db:(“LILACS”))	1369
PUBMED	(Infant OR premature [All Fields]) AND “home care” AND “maternal care pattern” [All Fields]	102
SCIELO	(“newborn premature” OR “newborn”) AND (“postnatal” OR “maternal behavior”) Filters applied: Not used.	124
Cochrane Library	Cochrane Reviews matching “premature newborn” in Title Abstract Keyword AND “ maternal care” in Title Abstract Keyword AND “patient discharge” in Title Abstract Keyword AND “home care” in Title Abstract Keyword AND “professional-family relationships” in Title Abstract Keyword - (Word variations have been searched)	0
Total		1612

### Quality Criteria for Selected Articles

The selection process for this integrative review identified and chose a sample of publications using two independent reviewers to minimize study biases. Additionally, articles with discrepancies between the two reviewers were assessed by a third, independent reviewer. Specifically, there were discrepancies in three articles during the initial review of titles, abstracts, and keywords. The third reviewer, considering the inclusion and exclusion criteria, decided to include these three articles in the comprehensive reading phase prior to the final selection.

### Study Selection

After making choices from the databases, the selection of the proposed study samples was exported to the Rayyan QCRI research tool. This tool is a multi-platform application available online and as a smartphone-compatible app. It’s a free, cloud-based tool designed to expedite the initial screening of titles, abstracts, and keywords through an automated and intuitive process, all while ensuring a high level of usability^(16)^.

The database search spanned from January to May of 2022, yielding a total of 1,612 articles. Of these, 1,198 articles were excluded due to duplication. Subsequently, 414 articles were evaluated based on their titles, abstracts, and keywords, following the inclusion and exclusion criteria. This led to a consolidated list of 100 articles. After a thorough reading, only 21 articles met the criteria and were chosen for this integrative review.

Two independent evaluators selected the studies, keeping the inclusion and exclusion criteria in mind. There were discrepancies in three articles; after discussion, the evaluators unanimously chose to include those three articles.

**Figure 1:**
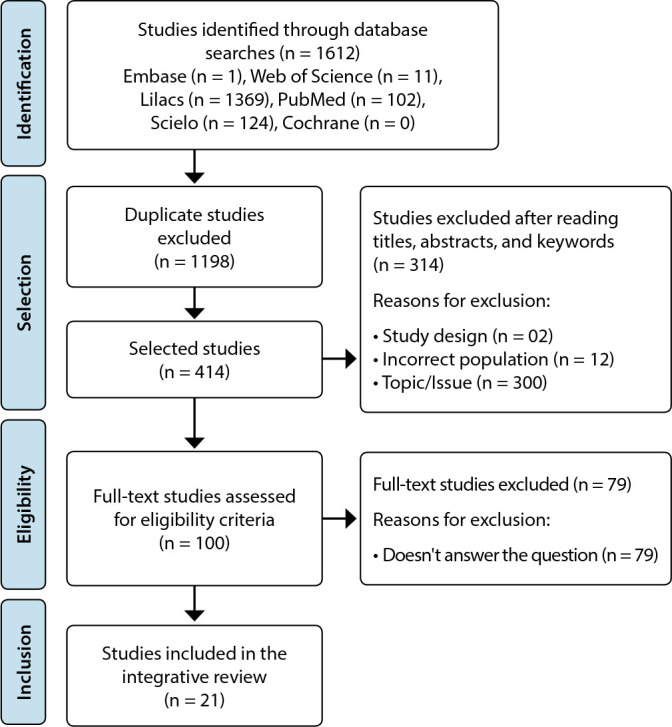
Flowchart illustrating the stages of identification, selection, and inclusion of studies, adapted from PRISMA^(17)^

Following this, we present the article selection process and detail the reasons for exclusions as per the PRISMA flowchart for Systematic Reviews^(17)^, modified for this Integrative Review (Figure 1). In this PRISMA flowchart adaptation, descriptive and heterogeneous articles were incorporated, even though they are typically omitted from systematic reviews.

After examining the 21 articles chosen for the selection, the subsequent details were retrieved: year, country, title, study type, level of evidence, and principal findings. This extraction utilized a form devised in alignment with the review’s objective and central question. To categorize the evidence level of the productions, the distinctions were made as follows:

Level I - Systematic review or meta-analysis of randomized or controlled clinical trials;

Level II - Evidence originating from at least one controlled and randomized clinical trial;

Level III - Non-randomized experimental clinical trials;

Level IV - Cohort or case-control studies;

Level V - Systematic reviews of descriptive and qualitative studies;

Level VI - Evidence stemming from descriptive or qualitative studies; and

Level VII - Evidence grounded in opinions from experts or specialized committees^(18)^.

## RESULTS

In this study, 21 articles from peer-reviewed journals were chosen and included. These articles were published in English and Portuguese from 2012 to 2021. The majority, five articles, were published in 2021, followed by three articles each in 2012 and 2020. The years 2013, 2014, 2017, and 2019 saw the publication of two articles each, and in both 2016 and 2018, one article was released. A significant portion (66.6%) of the articles originated from Brazil, whereas 33.3% came from other countries including Canada, Colombia, the United States, Iran, Italy, and Ghana. Predominantly, the articles in this review were descriptive studies with a qualitative approach and were classified under the sixth evidence level (Chart 2).

**Chart 2:** Overview of characteristics identified and extracted from the included articles in the study (N = 21)

Title/Reference	Year/ Country	Design	Outcome	Level of Evidence
*Recém-nascido prematuro: suporte materno domiciliar para o cuidado* ^(19)^	2012 Brazil	Descriptive Study	At home, mothers of premature newborns had questions about handling colic, the number of bowel movements, and sun exposure. To address their uncertainties and overcome feelings of fear and insecurity in performing certain tasks, they sought health professionals and consulted the guidebook provided at the time of hospital discharge.	VI
Gaining confidence and perspective: a phenomenological study of mothers’ lived experiences caring for infants at home after neonatal unit discharge^(20)^	2012 Canada	Phenomenological Study	In the home setting, mothers were anxious about the fragile health of their premature infants and were concerned about their well-being. Using instinct and a trial-and-error approach, they tried to identify their baby’s needs and signs of pain or discomfort.	VI
*Experiência materna no cuidado domiciliar ao recém-nascido prematuro* ^(21)^	2012 Brazil	Qualitative Study	For home care, mothers of premature infants expressed the need for guidance on child development, breathing, vaccination, breastfeeding, and the preparation and administration of medications.	VI
*Alta hospitalar e o cuidado do recém-nascido prematuro no domicílio: vivência materna* ^(22)^	2013 Brazil	Qualitative Study	The mothers’ experiences during the first week of home care following the hospital discharge of their premature newborns were marked by questions about bathing, signs of sickness, and other information not provided by professionals upon discharge.	VI
*Prematuro: Experiência materna durante amamentação em unidade de terapia intensiva neonatal e pós-alta* ^(23)^	2013 Brazil	Qualitative Study	At home, mothers had questions about continuing breastfeeding and the subsequent feeding regimen for the premature newborn.	VI
Caring for premature child at home: from fear and doubt to trust^(24)^	2014 Colombia	Phenomenological Study	The first night that mothers spent at home caring for their child was long, filled with anguish and uncertainties. By the next morning, they felt exhausted, filled with questions and concerns, fearing that their child might have lost weight or developed a complication that would require re-admission.	VI
*Ações de enfermagem na assistência domiciliar ao recém-nascido de muito baixo peso* ^(25)^	2014 Brazil	Qualitative Study	Mothers’ concerns during the daily care of the premature baby at home centered around hygiene, comfort, and recognizing signs and symptoms that appeared after the hospital discharge.	VI
Life after discharge: what parents of preterm infants say about their transition to home^(26)^	2016 USA	Qualitative Study	Uncertainties about breastfeeding, caring for the premature newborn at home without professional supervision, and finding reliable information sources led to stress, worry, and anxiety.	VI
*Preparing for post-discharge care of premature infants: Experiences of parents* ^(27)^	2017 Colombia	Qualitative Study	The findings emphasize the importance of creating institutional follow-up programs to ease the transition of the premature baby to the home setting. The value of family follow-up and the ability to contact the healthcare unit by phone for queries were highlighted as beneficial aspects of home care.	VI
Theory of infants’ transition management from then neonatal intensive care unit to home: a qualitative study^(28)^	2017 Iran	Qualitative Study - Grounded Theory	Typically, mothers of premature infants are not prepared to care for their children immediately postpartum. Once home, the mothers’ primary concern was the survival of their premature child, who had previously been under professional supervision in the hospital.	VI
*Percepção das mães quanto à competência materna nos cuidados domiciliares do recém-nascido prematuro* ^(29)^	2018 Brazil	Phenomenological Study	It was found that mothers of premature newborns have insufficient knowledge for home care of their child, especially when without the assistance of a healthcare professional, leading to feelings of fear and insecurity even in the face of common newborn complications.	VI
*Aleitamento materno exclusivo de prematuros e motivos para sua interrupção no primeiro mês pós-alta hospitalar* ^(30)^	2019 Brazil	Cross-sectional Study	It was observed that the lack of guidance for the mother might be related to the weaning of the premature newborn after hospital discharge.	VI
*Dúvidas maternas na alta hospitalar do recém-nascido* ^(31)^	2019 Brazil	Quantitative Descriptive Study	The majority of mothers stated they were not prepared to care for their child (60.1%). The most frequently mentioned uncertainties were about bathing (60%) and breastfeeding (56.7%).	VI
*Vivência materna com o Método Canguru no domicílio* ^(32)^	2020 Brazil	Qualitative Study	Mothers experienced feelings of fear and insecurity during the home phase of the Kangaroo Method and mentioned a lack of guidance provided by health professionals. They face daily challenges, such as temperature control of the premature newborn, breastfeeding, identifying colic, and the importance of the Kangaroo position at home.	VI
*Oportunidades de cuidados à criança prematura: visita domiciliar e suporte telefônico* ^(33)^	2020 Brazil	Qualitative Study	Mothers’ questions regarding care included feeding, bathing, diaper changing, stool characteristics, clinical changes, referrals to specialists, and the need for tests.	VI
*Vulnerabilidades para a criança prematura:contextos domiciliar e institucional* ^(34)^	2020 Brazil	Qualitative Study	Home experiences with the premature baby led to concerns related to breastfeeding, signs of clinical changes, and fears of possible re-hospitalization.	VI
Mothers’ experiences of caring for preterm babies at home: qualitative insights from an urban setting in a middle-income country^(35)^	2021 Ghana	Qualitative Study	Mothers expressed uncertainties about feeding the premature baby, temperature control, infection prevention, and reported difficulties in balancing child care with household chores.	VI
*Conhecimento de mães sobre cuidados aos recém-nascidos prematuros e aplicação do Método Canguru no domicílio* ^(36)^	2021 Brazil	Qualitative Study	There were observed strengths and weaknesses in the care of the premature newborn and in the implementation of the Kangaroo Method at home. The main questions from mothers were about warning signs for complications.	VI
A Study of Maternal Competence in Preterm Birth Condition, during the Transition from Hospital to Home: An Early Intervention Program’s Proposal^(37)^	2021 Italy	Quantitative Study	There was a trend towards undermining maternal competence in terms of the ability to care for the premature newborn in the post-discharge period. When mothers realize they don’t understand the true meaning of their child’s signals, an essential condition to feel confident in seeking solutions and making decisions, they can become extremely distressed.	IV
*Demandas de aprendizagem de famílias sobre cuidados pós-natais aos recém-nascidos* ^(38)^	2021 Brazil	Qualitative Study	There were evident misunderstandings and incorrect practices related to intimate hygiene, diaper changing, bathing, cleaning of the umbilical stump, use of products on the premature baby’s skin, and breastfeeding.	VI
*Cuidados com o recém-nascido prematuro após a alta hospitalar: investigação das demandas familiares* ^(39)^	2021 Brazil	Qualitative Study	Questions from mothers and relatives were highlighted concerning feeding, hygiene, sleep, and the presence of clinical changes.	VI

The depiction of the articles selected for this study, accounting for data extraction details like year, country, title, type of study, evidence level, and primary outcomes, can be found in Chart 2.

Regarding the main results, all the articles highlighted the primary questions and guidance needs that mothers of premature newborns experienced upon arriving home after hospital discharge. These concerns related to breastfeeding^(21,23-24,26,30-35,38-39)^, hygiene care^(22,25,31,33,38-39)^, sunbathing^(19)^, baby colic^(19,32)^, identification of signs, symptoms, or clinical changes^(20-22,25,29,33-35,38)^, temperature control^(32,35)^, and the continuity of the kangaroo method at home^(32,36)^.

Furthermore, the arrival of a premature child at home represents a critical adaptation period for the family. The care mothers provide is filled with feelings of anxiety, fear, and insecurity^(19,26,29-30,32)^, as well as concerns about potential re-hospitalization^(24,34)^ and the child’s survival^(28-29)^.

## DISCUSSION

Most studies in this integrative literature review that met the inclusion criteria were conducted in Brazil, with evidence obtained through a qualitative research design. While there is a governmental focus on establishing guidelines and objectives for comprehensive and compassionate care for critically or potentially ill newborns emphasizing human rights protection, comprehensive care, and encouragement of parental involvement^(40)^ it is notable that the scientific output regarding maternal concerns or needs arising post-discharge of premature newborns from 2012 to 2021 remains limited.

Parents eagerly anticipate their child’s discharge from the hospital. However, a premature birth can often postpone this due to the baby’s physiological immaturity and clinical instability, which may result in the premature neonate’s admission to an intensive care unit^(31)^. The child’s transition from the hospital to home can be fraught with anticipation and anxiety for the parents. Therefore, it is essential for healthcare professionals to provide support to ensure parents feel secure and prepared to continue caring for their premature child at home^(23,31-32)^.

Although some care instructions and demonstrations by professionals during hospitalization might seem straightforward, they can become challenging when implemented at home. This can make mothers and/or family members feel unprepared^(26,28,30)^, leading them to seek information from friends, relatives^(29,32)^, manuals^(19)^, or even online^(23)^.

At home, it is common for mothers to express concerns and feelings of fear, anxiety, and insecurity, among others^(19,24,26,28-29,32)^, especially regarding the care of the preterm baby when they lack immediate access to a multidisciplinary team for guidance and to address daily issues^(28-29,34,37)^.

The primary maternal concerns about caring for the premature newborn at home, as highlighted in the analyzed studies, focused on issues related to breastfeeding, hygiene, sunbathing practices, addressing baby colic, recognizing signs, symptoms, or clinical changes, ensuring thermal control, and maintaining the kangaroo method at home.

Concerning breastfeeding practices at home, mothers may face challenges such as: breast issues, achieving correct latching, managing the return to work, and recognizing satiety signs in their baby. If these challenges are not addressed with professional guidance or support from members of the mother’s social network, it could disrupt breastfeeding^(10,22,24-25,29,34,36)^. Factors leading to early weaning aren’t solely biological but also social, often arising from inadequate guidance and a lack of encouragement^(24)^. Moreover, using milk supplements both in hospital and at home, as well as a decrease in maternal milk supply, might relate to external influences, maternal fatigue, or fears of re-hospitalization due to the newborn’s insufficient weight gain^(24-25)^.

Regarding hygiene for the preterm neonate, mothers often have questions about the appropriate water temperature for baths, the products safe to use during bathing, the frequency of baths, diaper changing routines, rash prevention, and umbilical cord care^(10,19,24-25,27,34,36)^. These queries highlight the importance of involving caregivers in daily care, even during the hospital stay.

Regarding hygiene care, mothers may fear breaking a limb of the baby or injuring the baby while cleaning the umbilical stump^(38)^. A study conducted in Brazil, published in 2020 with the participation of 247 postpartum women aged between 20 and 34, revealed that 57.6% felt insecure about holding the baby due to its size. Additionally, 35.9% reported difficulties in holding the baby in the bathtub, 30.4% expressed fear of washing the baby’s back and genitals, 21.7% had questions about cleaning the baby’s head and face, and 12% had concerns about drying the neonate^(9)^.

Concerning sunbathing practices, it’s essential to emphasize the importance of proper guidance during hospitalization. In the first six months of a preterm baby’s life, exposure to sunlight should be indirect, using protective barriers such as hats, umbrellas, and clothes, due to the baby’s immature skin^(41)^.

Therefore, just as direct exposure to sunlight should be avoided, maintaining the neonate’s thermal control should be a priority for caregivers. Keeping the appropriate temperature for preterm babies is crucial because of the elevated risk of hypothermia. This risk arises specifically from inadequate heat regulation due to various organic factors, predominantly stemming from central immaturity in thermoregulatory control^(42-43)^.

As such, caregivers should be guided to maintain the body temperature of the preterm baby between 36.5ºC and 37.5ºC at home, using a thermometer placed in the armpit. This practice is essential for the neonate’s proper growth and development, preventing health complications that can result from a low body temperature, such as hypoglycemia, hypoxia, neonatal infections, and asphyxia^(42-43)^.

Similarly, addressing colic is essential, given that the gastrointestinal immaturity caused by premature birth can lead to episodes of diarrhea, vomiting, and severe colic. These symptoms can be attributed to delayed gastric emptying due to immature transient functions like entero-hepatic and decreased gastrointestinal motility^(44)^.

Hence, during hospitalization, it’s vital to emphasize the correct procedures in managing this condition, thereby preventing the use of empirical pharmacological interventions^(45)^, which in some cases might be unnecessary. Moreover, it should be reiterated that this is a natural occurrence, expected, and can be managed through breastfeeding and alleviated by providing emotional support to the mother-infant pair^(44)^.

Although studies indicate that upon hospital discharge, families receive guidance on the child’s overall health, factors such as recognizing signs and symptoms, discerning the reasons behind the baby’s crying, and noting clinical changes continue to be sources of uncertainty for mothers at home^(11,21-22,25,27,29,32-39)^.

From this viewpoint, combined fears of potential issues with the baby, limited knowledge about clinical changes, and maternal inexperience might prompt caregivers to adopt unsuitable practices. This could contribute to a rise in health complications for prematurely born children^(27)^.

This fact highlights the need for access to educational materials to which mothers can refer during moments of doubt, materials that are adapted to identify signs that might be considered clinical alterations^(22,30)^. Moreover, it’s of great importance that the healthcare team establishes a continuous follow-up program for these mothers, either in person or by phone^(37)^.

The increase in morbidities can follow a child born prematurely throughout their life. One strategy to reduce this issue is the Kangaroo Method^(40)^. Established in 1979, it contributed to the reduction of premature mortality in neonatal units through skin-to-skin contact and the strengthening of the emotional bond between mother and baby. In Brazil, it was introduced in the 1990s and incorporated into public health policies, becoming a governmental policy of the Ministry of Health^(30)^.

Currently, the use of the method in its three stages has been widely applied in the context of public health with positive impacts concerning the promotion of breastfeeding, reduction of health complications for the preterm neonate, and the support of growth and development^(40)^. However, the significant challenge is the adherence to this method outside the hospital environment^(40,46)^, that is, at home, where challenges and doubts can arise^(10,32,35)^. Therefore, it’s essential that these mothers are familiar with the position and understand its importance during their stay in the hospital, something that should be encouraged and reinforced by the entire professional team. This way, after being discharged, they can confidently apply the method at home^(30)^.

In this regard, it can be observed that discontinuation of the method is often related to the mother’s lack of knowledge about its benefits and their own insecurity in applying it without professional support. Additionally, the low application can also be associated with a lack of encouragement from the team during their stay in the unit^(10,30)^.

When the family arrives home with a premature neonate, feelings of anxiety, fear, and insecurity intensify in the face of the daily demands of special care for the baby. It’s worth noting that this care, previously carried out by a professional with the assistance of a caregiver, now falls solely on the family members^(11,19,26,29,34,37)^. Added to this insecurity, mothers might experience feelings of apprehension or concern about their child’s health, mainly for fear that prematurity may result in lasting effects, increasing the risk of illness, rehospitalization, or even death under their care^(20,23-24,28,32)^.

In this context, it’s clear that there’s still a disconnect between what is advised by health professionals at the time of discharge and the reality of home care. This emphasizes the urgency of strategies that ensure the continuity of care for premature neonates and their families in the home environment^(27)^.

### Study Limitations

A significant limitation of this study is the limited number of articles from countries outside Brazil, identified in the databases using the specified search terms. Additionally, the majority of these studies are of a descriptive-qualitative nature. Despite these limitations, we are confident that the quality of our research remains intact. The results underscore the significance of future studies intended to expand discussions on the care of prematurely born children, especially concerning maternal needs that might emerge in a home setting.

### Contributions to Nursing and Health

Given the above, it’s crucial to emphasize the importance of the nursing team working in unison with other healthcare professionals. By approaching care from an interdisciplinary perspective, the focus remains on providing comprehensive care to premature infants, whether in medical facilities or at home. In doing so, it’s possible to offer both nurturing and practical support to mothers grappling with concerns and the emotional challenges that come from caring for a newborn at home. In this scenario, the role of primary healthcare professionals is pivotal. They bear the responsibility for ongoing care after hospital discharge, and their support is invaluable in assisting mothers, thus making family adaptation to neonatal care at home smoother.

## CONCLUSIONS

This integrative literature review shines a light on the knowledge surrounding primary maternal concerns when caring for prematurely born infants at home. The studies reviewed were consistent in their responses to the central inquiry, highlighting that upon returning home with a premature newborn, the primary uncertainties mothers face pertain to breastfeeding, hygiene, sunbathing practices, addressing baby’s colic, recognizing signs, symptoms, or clinical changes, maintaining thermal control, and the consistent application of the kangaroo care method. Beyond the specifics of neonatal care, a mother’s daily life is deeply influenced by feelings of anxiety, fear, insecurity, and concerns. This emotional backdrop often makes mothers feel ill-prepared to continue caring for their premature infant at home, even after receiving thorough instructions from healthcare professionals upon hospital discharge.

This insight into maternal challenges accentuates the imperative nature of refining strategies that bolster family support and guarantee ongoing care for newborns at home. The scarcity of both national and international publications addressing this subject emphasizes the urgency of further research. This research should span a broad spectrum of scientific rigor, including randomized controlled clinical trials, case-control or cohort studies, other systematic reviews, and deep dives into critically evaluating the efficacy of healthcare professionals’ responses to the daily challenges and needs faced by families with a prematurely born child.
